# The nuclei of the lateral lemniscus: unexpected players in the descending auditory pathway

**DOI:** 10.3389/fnana.2023.1242245

**Published:** 2023-08-09

**Authors:** Mario Gómez-Martínez, Héctor Rincón, Marcelo Gómez-Álvarez, Ricardo Gómez-Nieto, Enrique Saldaña

**Affiliations:** ^1^Neuroscience Institute of Castilla y León, University of Salamanca, Salamanca, Spain; ^2^Department of Cell Biology and Pathology, Medical School, University of Salamanca, Salamanca, Spain; ^3^Institute of Biomedical Research of Salamanca, Salamanca, Spain

**Keywords:** superior olivary complex, medial nucleus of the trapezoid body (MNTB), ventral nucleus of the trapezoid body (VNTB), superior paraolivary nucleus (SPON), semilunar nucleus, paralemniscal, FluoroGold (FG), biotinylated dextran amine (BDA)

## Abstract

**Introduction:**

In the mammalian auditory pathway, the nuclei of the lateral lemniscus (NLL) are thought to be exclusively involved in the bottom-up transmission of auditory information. However, our repeated observation of numerous NLL neurons labeled after injection of retrograde tracers into the superior olivary complex (SOC) led us to systematically investigate with retrograde tracers the descending projections from the NLL to the SOC of the rat.

**Methods:**

We performed large injections of FluoroGold into the SOC to determine NLL contributions to descending projections, and focal injections of biotinylated dextran amine (BDA) to pinpoint the specific nuclei of the SOC innervated by each NLL.

**Results:**

The SOC is innervated by thousands of neurons distributed across four nuclei or regions associated with the lateral lemniscus: the ipsilateral ventral and intermediate nuclei of the lateral lemniscus (VNLL and INLL); the medial paralemniscal region (PL) of both sides; and the ipsilateral semilunar nucleus (SLN), a previously unrecognized nucleus that wraps around the INLL dorsally, medially, and caudally and consists of small, flat neurons. In some experiments, at least 30% of neurons in the VNLL and INLL were retrogradely labeled. All nuclei of the SOC, except the medial and lateral superior olives, are innervated by abundant lemniscal neurons, and each SOC nucleus receives a unique combination of lemniscal inputs. The primary target of the projections from the VNLL is the ventral nucleus of the trapezoid body (VNTB), followed by the superior paraolivary nucleus (SPON), and the medial nucleus of the trapezoid body (MNTB). The INLL selectively innervates the VNTB. The PL innervates dorsal periolivary regions bilaterally. The SLN preferentially innervates the MNTB and may provide the first identified non-calyceal excitatory input to MNTB neurons.

**Discussion:**

Our novel findings have strong implications for understanding acoustic information processing in the initial stages of the auditory pathway. Based on the proportion of lemniscal neurons involved in all the projections described, the NLL should be considered major players in the descending auditory pathway.

## 1. Introduction

Animals are continuously exposed to complex sensory contexts. To cope efficiently with overwhelming inputs, the nervous system has developed modulatory mechanisms that process and filter incoming information to enhance its most salient aspects while ignoring irrelevant stimuli. The processing of acoustic stimuli is modulated by feedback pathways that adjust feedforward signals in a context-dependent manner (Asilador and Llano, 2021; Lesicko and Geffen, 2022; Lesicko et al., 2022).

In mammals, the descending auditory pathway, which extends from the cerebral cortex to the organ of Corti, consists of complex arrays of long direct projections, polysynaptic chains and loops. First, through long-range corticosubcollicular projections, the auditory cerebral cortex directly regulates the activity of the earliest stages of auditory processing [reviewed by Saldaña (2015)]. Second, the descending pathway can be viewed as a cascading oligosynaptic chain with three main steps or links: the cerebral cortex sends massive projections to the inferior colliculus (IC) —corticocollicular link— [reviewed by Stebbings et al. (2014)], which in turn innervates the superior olivary complex (SOC) —colliculo-olivary link— (Suthakar and Ryugo, 2017; Romero and Trussell, 2021), whose medial olivocochlear neurons innervate hair cells in the organ of Corti —olivocochlear link— [reviewed by Guinan (2018), Lopez-Poveda (2018), and Lauer et al. (2022)]. Finally, the descending auditory pathway includes numerous local or regional loops, whereby the activity of specific auditory nuclei is modulated by top-down projections from higher centers innervated by them. Some of the most dense and well-characterized circuits include the corticothalamic loop [reviewed by Antunes and Malmierca (2021)], the colliculo-olivary loop (Cant and Benson, 2006; Cant, 2013; Romero and Trussell, 2021), and the colliculo-cochleonuclear loop (Malmierca et al., 2005; Milinkeviciute et al., 2017; Balmer and Trussell, 2022).

Thus, all stations of the mammalian auditory pathway are known to be involved in the descending system, with one notable exception: the nuclei of the lateral lemniscus (NLL). The three classical NLL (ventral [VNLL], intermediate [INLL], and dorsal [DNLL]) form a column of gray matter located in the lateral aspect of the brainstem tegmentum interspersed among the fascicles of the lateral lemniscus. The NLL do not receive projections from higher centers. Moreover, most, if not all, neurons in the VNLL and INLL have been reported to innervate the ipsilateral IC (Saint Marie and Baker, 1990; Merchán and Berbel, 1996; Zimmerman et al., 2020), and DNLL neurons send their axons to either the ipsilateral or the contralateral IC (Shneiderman et al., 1993; Merchán et al., 1994; Kelly et al., 2009; Zimmerman et al., 2020). These facts have led to the general assumption that the NLL are mainly, if not exclusively, involved in the bottom-up transmission of auditory information.

During our hodological investigations of the auditory pathway of rodents, we noticed that injections of retrograde tracers into the SOC resulted in numerous labeled neurons in the region of the lateral lemniscus, particularly within the VNLL. This was not an infrequent or sporadic observation, but rather a consistent finding that challenged the dogma of the NLL as relays in the ascending auditory pathway whose neurons do not give rise to descending projections. Because this finding could have strong implications for understanding acoustic information processing in the initial stages of the auditory pathway, we set forth to systematically investigate with tract-tracing techniques the extent to which the NLL and their surrounding territories contribute to the innervation of the SOC. More specifically, we have analyzed qualitatively and quantitatively the lemniscal neurons that innervate each of the SOC nuclei.

## 2. Materials and methods

### 2.1. Experimental design

In keeping with the commitment of the scientific community to decrease the use of experimental animals in biomedical research through the “three Rs” (reduce, refine, and replace), we have undertaken this study by resorting to a fourth “R”: reuse. Accordingly, this investigation is mostly based on our large collection of cases with single injections of retrograde or bidirectional tracers into the SOC of rodents, obtained over more than twenty-five years. To increase the number of cases in some of the experimental groups, we have recently performed some *ad hoc* experiments (*n* = 3). The procedures applied to these additional cases were essentially identical to those used in the archival cases, many of which are described in the publications mentioned below.

We performed our study in two steps. First, to determine the extent to which the classical NLL and their surrounding territories contain neurons that innervate the SOC, we examined 10 cases with single injections of the retrograde tracer FluoroGold that affected sizable portions of the rat SOC. Seven of these cases were used in previous studies of the connections of the SOC (Saldaña et al., 2007; Marshall et al., 2008). As shown below, these experiments revealed an unexpectedly large number of neurons labeled in the region of the lateral lemniscus of both sides. Second, to identify the specific nuclei of the SOC innervated by these neurons, we analyzed 47 cases with small, single injections of the bidirectional tracer BDA into the rat SOC. Out of our large collection of cases, we selected those in which the injection site was wholly confined to a single nucleus of the SOC or minimally affected adjacent structures. Twenty one of these cases had been used in previous studies of connections of SOC nuclei (Gómez-Nieto et al., 2008, 2014; Saldaña et al., 2009; Viñuela et al., 2011; Gómez-Álvarez and Saldaña, 2016).

### 2.2. Experimental animals

The rats used in our experiments were all young adult female albino specimens (Rattus norvegicus, Wistar or Sprague-Dawley strains; body weight 190–210 g) obtained from the Animal Core Facility of the University of Salamanca or from Charles River France. All the animals were cared for and used in compliance with Spanish and European Union regulations on the use of animals in biomedical research. The experimental procedures were approved and supervised by the Bioethics Committee of the University of Salamanca (permits associated with grants DGICYT PB97-1326, CyL SA 15/97, FIS 97/0866, CyL SA 27/99, FIS 99/1186, BFI2000-1358, CyL SA079/01, BFU2004-05909, PI10/01803, CyL SA007C05, BFU2008-04197 and CyL GR221).

For the surgical injection of neuroanatomical tracers, the animals were deeply anesthetized with a mixture of ketamine HCl (80 mg/kg body weight) and xylazine (6 mg/kg body weight) administered intramuscularly or intraperitoneally. Postoperative pain relief was provided by subcutaneous injection of buprenorphine (0.05 mg/kg body weight) every 12 h for 3 days. For the transcardial perfusion of fixatives, the animals were anesthetized with the same mixture of ketamine HCl and xylazine used for surgery or, more often, with an overdose of sodium pentobarbital administered intraperitoneally.

### 2.3. Tract-tracing with FluoroGold

We injected the retrograde fluorescent tracer FluoroGold (Fluorochrome Inc., Denver, CO, USA) as a 4% solution in saline. Under stereotaxic guidance, tracer-loaded glass micropipettes (20–40 μm inner diameter at the tip) were lowered into the right SOC. To avoid damage to the prominent transverse sinus, in most cases the pipettes were lowered into the brain via a dorsocaudal to ventrorostral approach, so that their trajectory formed a 16–20° angle with the coronal plane. The tracer was delivered by iontophoresis using a pulsed 5 μA DC positive current (7 s on/7 s off) for 5–15 min. The current was then stopped, and the pipette left in place for another 5–20 min before withdrawal to prevent tracer leakage along the injection tract. Animal suffering was minimized by monitoring the depth of anesthesia using physiological cues such as rate and depth of breathing and reflex response to toe pinch. Supplemental doses of anesthetics were administered as needed to ensure deep anesthesia throughout the procedure.

After 7 days of survival, the rats were again anesthetized, and their brains were fixed by transcardial perfusion with buffered 4% formaldehyde, prepared from commercial formalin or from freshly depolymerized paraformaldehyde. The brains were carefully dissected, and an entomological needle (size 000, diameter 0.25 mm) was inserted longitudinally on the side contralateral to the injection site. The fiducial mark left by the needle was later used to identify the left and right sides of each section, and to achieve an accurate dorsoventral alignment of the sections. After cryoprotection in 30% sucrose in phosphate buffer, the brains were sectioned at 40–50 μm thickness on a freezing microtome. Most brains were cut coronally.

The fluorescent tracer FluoroGold was rendered permanently visible by immunocytochemistry on free-floating sections, using a rabbit anti-FluoroGold primary antiserum (Chemicon International, Inc; Temecula, CA, USA; 1:4,000), followed by biotinylated goat anti-rabbit immunoglobulin G (Vector Labs., Burlingame, CA, USA; 1:50). The sections were then processed by the avidin-biotin-peroxidase complex procedure following the manufacturer’s specifications (ABC, Vectastain, Vector), and then by histochemistry for peroxidase using 3-3′-diaminobenzidine tetrahydrochloride hydrate (DAB; Sigma, product D5637; Saint Louis, MO, USA) as the chromogen. A percentage of the sections were reacted in a mixture containing 0.025% DAB and 0.00015% H_2_O_2_ in 0.05 M tris buffer, at pH 7.6, which results in the standard brown reaction product. Most sections, however, underwent a nickel-enhanced DAB reaction, which produces a dark blue/ black reaction product; to this end, they were incubated in a mixture containing 0.015% DAB, 0.4% nickel (II) ammonium sulfate hexahydrate (Carlo Erba, product 464545; Milano, Italy), and 0.00015% H_2_O_2_ in 0.05 M tris buffer, at pH 8.0. Following the histochemical reaction, the sections were mounted on gelatin-coated slides, air dried, dehydrated, cleared, and coverslipped with Entellan^®^ New (Merck Millipore, product 1.07961.0100; Darmstadt, Germany). For cytoarchitectural reference, a percentage of the sections—in most cases, every fourth section—was counterstained with cresyl violet before coverslipping.

### 2.4. Tract-tracing with BDA

Glass micropipettes (10–20 μm inner tip diameter) loaded with the bidirectional tracer BDA (biotinylated dextran amine, 10,000 MW, Molecular Probes, Eugene, OR, USA; 10% in 0.1 M sodium phosphate buffer, pH 7.4) were stereotaxically guided into the SOC. The stereotaxic technique, iontophoretic tracer delivery, survival time, brain fixation by transcardial perfusion, and brain sectioning were as described above.

To visualize the tracer, the sections were first incubated in the avidin-biotin-peroxidase complex, and then processed by standard histochemistry for peroxidase, with heavy-metal intensification, as described above. For cytoarchitectural reference, every fourth section was counterstained with cresyl violet.

### 2.5. Cytoarchitectural references

#### 2.5.1. Topography and parcellation of the SOC

**FIGURE 1:**
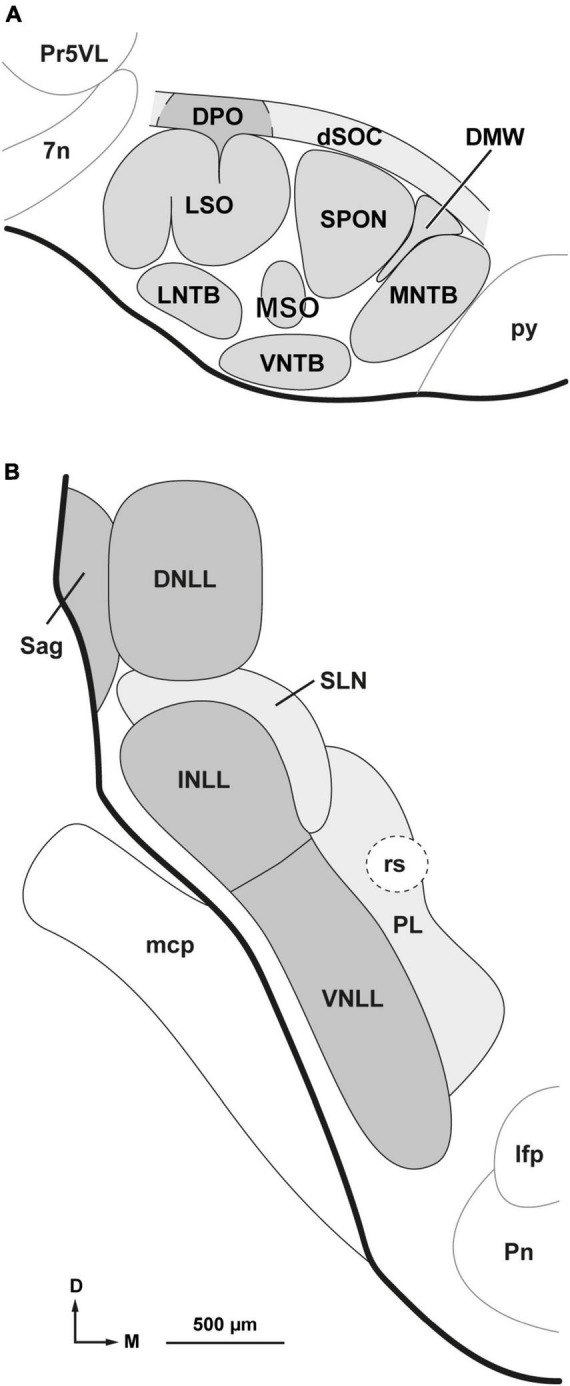
Parcellation of the superior olivary complex (SOC) and nuclei of the lateral lemniscus (NLL). **(A)** Schematic representation of the parcellation of the rat SOC as it appears in coronal sections through the caudal half of the complex. In addition to the six classical nuclei (lateral and medial superior olives [LSO and MSO, respectively], superior paraolivary nucleus [SPON], and medial, ventral and lateral nuclei of the trapezoid body [MNTB, VNTB and LNTB, respectively]), the complex includes: the dorsomedial wedge (DMW), sandwiched between SPON and MNTB; the dorsal ribbon (dSOC), located dorsal to SPON, DMW, and MNTB; and the dorsal periolivary nucleus (DPO), located above LSO. **(B)** Schematic representation of the parcellation of the NLL as they appear in coronal sections through their mid-rostrocaudal level. In the rat, there are six NLL: the three classical nuclei (ventral [VNLL], intermediate [INLL] and dorsal [DNLL]); the nucleus sagulum (Sag), which lies lateral to the DNLL; the previously unrecognized semilunar nucleus (SLN), which covers the INLL medially, dorsally and caudally; and the medial paralemniscal region (PL), which lies medial to the VNLL and SLN. Other abbreviations: 7n, root of the facial nerve; lfp, longitudinal fascicle of the pons; mcp, middle cerebellar peduncle; Pn, pontine nuclei; Pr5VL, principal sensory trigeminal nucleus, ventrolateral part; py, pyramidal tract; rs, rubrospinal tract. The calibration bar in **(B)**, uncorrected for shrinkage, also applies to **(A)**.

To accurately characterize the location of each injection site within the SOC, we elaborated a model of the parcellation of this complex. In coronal sections through the caudal half of the SOC, the complex consists of nine distinct nuclei or cell groups (highlighted in gray in Figure 1A). Six of these SOC components are well-characterized nuclei recognized in most previous studies in rats: lateral superior olive (LSO), medial superior olive (MSO), superior paraolivary nucleus (SPON), medial nucleus of the trapezoid body (MNTB), lateral nucleus of the trapezoid body (LNTB), and ventral nucleus of the trapezoid body (VNTB) (Harrison and Feldman, 1970; Osen et al., 1984; Vetter et al., 1991; Friauf, 1993; Sommer et al., 1993; Lohmann and Friauf, 1996; Warr et al., 1997; Kulesza and Berrebi, 2000; Saldaña and Berrebi, 2000; Kulesza et al., 2002; Doucet and Ryugo, 2003; Kelly et al., 2009; Ito et al., 2011; Mansour and Kulesza, 2021).

The SOC also includes three other subdivisions that are relevant to the present investigation:

The dorsal ribbon of the SOC (dSOC): the dSOC was identified by Feliciano et al. (1995) as a narrow and ill-defined region that overlays the SOC dorsally and receives direct projections from the cerebral auditory cortex of both sides. This flattened structure covers the dorsal aspect of the SPON, the dorsomedial wedge (DMW, see below) and the MNTB, and is approximately 200 μm thick dorsoventrally.

The dorsal periolivary nucleus (DPO): the DPO is an ill-defined structure located just dorsal to the LSO (up to 200 μm from its dorsal limit) and lateral to the dSOC. Although its defining features have not been standardized, the DPO differs from its surrounding territories in that it receives dense projections from octopus neurons of the PVCN (Warr, 1969; Adams and Warr, 1976; Friauf and Ostwald, 1988), contains shell olivocochlear neurons (Vetter and Mugnaini, 1992; Warr et al., 1997; Sánchez-González et al., 2003), and contains a rich network of fibers and cell bodies that express the neuromodulatory peptide urocortin-3 (Fischl et al., 2019).

The dorsomedial wedge (DMW): this cell group occupies the angle between the dorsomedial border of the SPON and the dorsolateral border of the MNTB. It was first identified by Saldaña and Berrebi and their coworkers as a discrete group of neurons that send direct projections to the auditory thalamus (Berrebi et al., 2012, 2013a, 2013b; Saldaña et al., 2012, 2013; Saldaña, 2013). The rat DMW contains approximately 2,450 neurons with medium-sized, predominantly round or oval cell bodies (Mansour et al., 2021). Evidence from immunostaining for glutamic acid decarboxylase and from *in situ* hybridization for the vesicular inhibitory amino acid transporter suggests that most, if not all, DMW neurons are GABAergic (Vetter et al., 1991 [see their Figure 1A]; Kulesza and Berrebi, 2000 [their Figures 5, 6]; Ito et al., 2011 [their Figure 6]).

#### 2.5.2. Topography and parcellation of the nuclei of the lateral lemniscus

In this study, we use the term nuclei of the lateral lemniscus (NLL) in a broad sense to refer not only to the three classical NLL (ventral, intermediate, and dorsal), but also to three other nuclei or territories adjacent to them and anatomically related to the lateral lemniscus: the nucleus sagulum, the semilunar nucleus and the medial paralemniscal region. Accordingly, we distinguish six NLL, explained below, and depicted schematically in Figure 1B.

The ventral nucleus of the lateral lemniscus (VNLL) and the intermediate nucleus of the lateral lemniscus (INLL) form a compact column of gray matter interspersed among the fiber fascicles of the ventral three-quarters of the lateral lemniscus. The VNLL and the INLL differ in several respects [thoroughly discussed by Nayagam et al. (2010), Felmy (2019), and García-Guillén et al. (2023)]. Although the boundary between the VNLL and the INLL is poorly defined, in the rat the transition between the two nuclei occurs approximately at the level of the dorsal border of the rubrospinal tract (Figure 1B).

The dorsal nucleus of the lateral lemniscus (DNLL) is a cytoarchitecturally distinct structure located between the INLL and the inferior colliculus (IC). It is traversed by the fiber bundles of the dorsal quarter of the lateral lemniscus and its features in the rat have been extensively described in previous studies (Bajo et al., 1993; Merchán et al., 1994; Saint Marie et al., 1999; Kelly et al., 2009).

The nucleus sagulum is wedged between the lateral border of the DNLL and the surface of the brainstem (Figure 1B). It consists mostly of small neurons with oval cell bodies that tend to be oriented parallel to the pial surface (Henkel and Shneiderman, 1988; Hutson et al., 1991; Bajo et al., 1993; Merchán et al., 1994; Beneyto et al., 1998).

As will become evident in the section of “3. Results,” the previously unrecognized semilunar nucleus (SLN) is a group or sheet of densely packed, predominantly small, flattened neurons with distinct projections. This narrow nucleus wraps around the INLL dorsally, medially, and caudally. In most coronal sections through the INLL, the shape of the SLN resembles a crescent or half-moon (Figure 1B), hence its name. The dorsal part of the SLN corresponds to the “horizontal cell group,” interposed between the DNLL and the INLL (Ruiz-Gómez, 1988; Bajo et al., 1993; Caicedo and Herbert, 1993; Merchán et al., 1994; Kulesza et al., 2002).

The medial paralemniscal region (PL) is an ill-defined, poorly celled territory located medial to the SLN and the VNLL (Figure 1B). It extends approximately 500 μm from the medial border of the VNLL and largely corresponds to the roughly vertical, narrow territory that receives projections from the ipsilateral primary auditory neocortex (Feliciano et al., 1995; Saldaña, 2015). It is crossed by the rubrospinal tract, which, in coronal sections, appears as a round patch devoid of neurons.

The portion of the PL dorsal to the rubrospinal tract partially overlaps with the so called medial paralemniscal nucleus, whose neurons express the tuberoinfundibular peptide of 39 residues (TIP39; Dobolyi et al., 2002, 2003a,b; Varga et al., 2008). We did not include this nucleus in our parcellation because, as discussed in later sections of this article, the medial paralemniscal nucleus does not appear to project to the SOC.

### 2.6. Data analysis

We first analyzed the sections qualitatively to describe the location of the labeled neurons in all the NLL defined below. Next, we characterized the morphology of the labeled neurons paying attention to criteria such as cell body size and shape, number of primary dendrites, dendritic branching, and orientation of the dendrites relative to the lemniscal fibers. Finally, we counted the labeled neurons in the NLL of all experimental cases included in this study.

#### 2.6.1. Number of labeled neurons

Using ImageJ software (version 1.53k), we counted every labeled neuron visible in high-resolution micrographs of all the sections containing the NLL. Micrographs were taken with a Zeiss Axioskop 40 microscope equipped with a Zeiss AxioCam MRc 5 digital camera (Carl Zeiss, Oberkochen, Germany). To correct for oversampling due to split neurons appearing in two adjacent sections, we applied Abercrombie’s formula (Abercrombie, 1946):


$$N=n\times \frac{T}{D+T}$$

where N is the estimated number of labeled neurons, n is the number of neurons counted, T is the section thickness, and D is the mean maximum diameter of the labeled cell bodies. To calculate the mean cell body diameter, we used the case with the most labeled neurons (case FG01) and measured the maximum diameter of every labeled neuron with sharp cell body contour found in each ipsilateral NLL (see Table 1 below). To measure the maximum diameter, we traced the contour of the cell body on high-resolution micrographs using the polygon tool of ImageJ.

**TABLE 1:** Average ± standard deviation (SD) of the maximum and minimum diameter of the cell body of NLL neurons retrogradely labeled following a large injection of FluoroGold into the ipsilateral SOC.

Nucleus	Number of measured neurons	Maximum diameter ± SD	Minimum diameter ± SD
Ventral nucleus of the lateral lemniscus (VNLL)	168	16.8 ± 3.8	10.8 ± 2.2
Intermediate nucleus of the lateral lemniscus (INLL)	73	15.9 ± 3.3	9.2 ± 2.0
Medial paralemniscal regions (PL)	70	16.0 ± 4.1	9.2 ± 2.5
Semilunar nucleus (SLN)	60	14.8 ± 4.1	8.2 ± 1.5
Dorsal nucleus of the lateral lemniscus (DNLL)	28	18.3 ± 4.7	11.4 ± 2.1
Sagulum	61	14.7 ± 3.3	8.8 ± 1.9

#### 2.6.2. Statistical analyses

We compared the size of retrogradely labeled neurons using a one-way ANOVA test. In addition, we classified our cases with small injections of BDA into eight groups defined by the SOC nucleus that received the tracer injection. We first analyzed the number of labeled neurons in each NLL using the Shapiro–Wilk normality test. For this analysis, we excluded the DNLL and the sagulum because in most cases the number of labeled neurons in these structures was negligible. The Shapiro–Wilk test showed that the number of neurons labeled in VNLL, INLL, medial paralemniscal region (PL) and SLN did not follow a normal distribution. We then applied the non-parametric Kruskal–Wallis test to compare the eight groups using the number of neurons labeled in the VNLL, INLL, PL, and SLN as variables. All statistical analyses were performed using IBM SPSS Statistics 21 software.

#### 2.6.3. Illustrations

To illustrate the neurons labeled in the NLL, we photographed the sections with the Zeiss AxioCam MRc 5 digital camera attached to a Zeiss Axioskop 40 microscope. We obtained panoramic images using a 5× (N.A. = 0.10), 10× (N.A. = 0.25) or 20× (N.A. = 0.40) objective lens. To illustrate individual labeled neurons, we captured high-resolution images of the same section at different focal planes with a 40× objective lens (N.A. = 0.75) or a 100× oil immersion objective lens (N.A. = 1.25). The images were then stacked and collapsed into one single, maximum focus image using Helicon Focus Pro software (HeliconSoft, Kharkiv, Ukraine). The brightness and contrast of the images were adjusted using Adobe Photoshop software 2020 (Adobe Systems, San Jose, CA, USA), and the illustrations were arranged into plates using Adobe Illustrator software 2020 (Adobe Systems). Adobe Illustrator was also used to draw the outline of the nuclei of the NLL and SOC, and to plot the neurons based on photographs of real sections. Although the tracer was always injected into the right SOC, all illustrations have been flipped horizontally, so that the side that received the injection site appears on the left.

## 3. Results

The results, obtained with albino rats, are divided into two parts. In the first, we present the results of experiments in which FluoroGold was injected into the SOC. The second part shows the results of experiments with small injections of biotinylated dextran amine (BDA) restricted to individual SOC nuclei.

### 3.1. Cases with injection of FluoroGold

These results are based on 10 cases with single injections of FluoroGold that affected sizable portions of the SOC and provided satisfactory filling of numerous retrogradely labeled neurons, including their cell body and proximal dendrites (Table 2).

**TABLE 2:** Number and percentage of neurons labeled retrogradely in each of the NLL following single injections of FluoroGold into the SOC.

	Ipsilateral							Contralateral							
Case number	VNLL	INLL	PL	SLN	Sag	DNLL	Total ipsi	VNLL	INLL	PL	SLN	Sag	DNLL	Total contra	TOTAL
**FG01**															
No. neurons	2,669	1,255	1,700	1,254	181	56	7,115	34	54	928	230	59	61	1,366	8,481
% own side	37.5%	17.6%	23.9%	17.6%	2.5%	0.8%	100%	2.5%	4%	67.9%	16.8%	4.3%	4.5%	100%	
% total	31.5%	14.8%	20%	14.8%	2.1%	0.7%	83.9%	0.4%	0.6%	10.9%	2.7%	0.7%	0.7%	16.1%	
**FG02**															
No. neurons	962	244	1,045	499	37	2	2,789	31	3	385	0	0	29	448	3,237
% own side	34.5%	8.7%	37.5%	17.9%	1.3%	0.1%	100%	6.9%	0.7%	85.9%	0%	0%	6.47%	100%	
% total	29.7%	7.5%	32.3%	15.4%	1.1%	0.1%	86.2%	1%	0.1%	11.9%	0%	0%	0.9%	13.8%	
**FG03**															
No. neurons	114	3	95	400	7	1	620	31	10	44	15	0	3	103	723
% own side	18.4%	0.5%	15.3%	64.5%	1.1%	0.2%	100%	30.1%	9.7%	42.7%	14.6%	0%	2.9%	100%	
% total	15.8%	0.4%	13.1%	55.3%	1%	0.1%	85.8%	4.3%	1.4%	6.1%	2.1%	0%	0.4%	14.2%	
**FG04**															
No. neurons	747	164	454	445	4	1	1,815	123	23	197	107	0	0	450	2,265
% own side	41.2%	9%	25%	24.5%	0.2%	0.1%	100%	27.3%	5.1%	43.8%	23.8%	0%	0%	100%	
% total	33%	7.2%	20%	19.6%	0.2%	< 0.1%	80.1%	5.4%	1%	8.7%	4.7%	0%	0%	19.9%	
**FG05**															
No. neurons	24	0	1,227	394	3	6	1,654	N.A.	N.A.	N.A.	N.A.	N.A.	N.A.	N.A.	N.A.
% own side	1.5%	0	74.2%	23.8%	0.2%	0.4%	100%								
**FG06**															
No. neurons	1,138	340	236	333	55	30	2,132	N.A.	N.A.	N.A.	N.A.	N.A.	N.A.	N.A.	N.A.
% own side	53.4%	15.9%	11.1%	15.6%	2.6%	1.4%	100%								
**FG07**															
No. neurons	824	412	123	491	36	12	1,898	N.A.	N.A.	N.A.	N.A.	N.A.	N.A.	N.A.	N.A.
% own side	43.4%	21.7%	6.5%	25.9%	1.9%	0.6%	100%								
**FG08**															
No. neurons	815	618	257	665	80	9	2,444	N.A.	N.A.	N.A.	N.A.	N.A.	N.A.	N.A.	N.A.
% own side	33.3%	25.3%	10.5%	27.2%	3.3%	0.4%	100%								
**FG09**															
No. neurons	497	154	248	365	20	9	1,293	24	4	71	46	1	1	147	1,440
% own side	38.4%	11.9%	19.2%	28.2%	1.5%	0.7%	100%	16.3%	2.7%	48.3%	31.3%	0.7%	0.7%	100%	
% total	34.5%	10.7%	17.2%	25.3%	1.4%	0.6%	89.8%	1.7%	0.3%	4.9%	3.2%	0.1%	0.1%	10.2%	
**FG10**															
No. neurons	1,449	355	679	529	46	11	3,069	21	11	180	38	4	10	264	3,333
% own side	47.2%	11.6%	22.1%	17.2%	1.5%	0.4%	100%	8%	4.2%	68.2%	14.4%	1.5%	3.8%	100%	
% total	43.5%	10.7%	20.4%	15.9%	1.4%	0.3%	92.1%	0.6%	0.3%	5.4%	1.1%	0.1%	0.3%	7.9%	

N.A., not available.

#### 3.1.1. Case with a large injection of FluoroGold into the SOC: case FG01

The injection site of case FG01 involved all nine nuclei of the SOC to some extent. This case had more retrogradely labeled neurons than any other case and lent itself to a detailed analysis of the number, distribution, and morphology of the labeled neurons.

##### 3.1.1.1. Injection site

**FIGURE 2:**
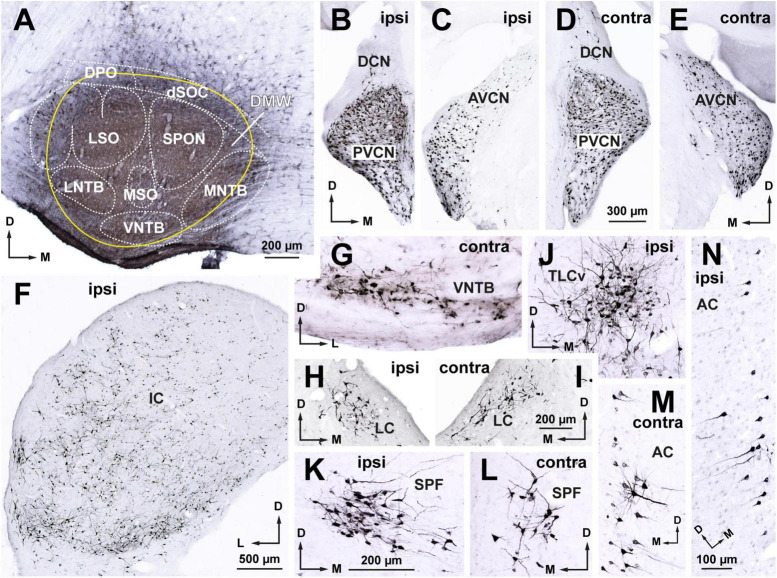
Following a large injection of FluoroGold into the rat superior olivary complex (SOC), abundant neurons are retrogradely labeled in all nuclei known to innervate the SOC. Micrographs of coronal sections from case FG01. **(A)** Section through the center of a large injection site of FluoroGold (outlined in yellow) that affected all nuclei of the SOC. **(B–E)** Neurons labeled in the ipsilateral **(B,C)** and contralateral **(D,E)** cochlear nuclei. Notice the abundance of labeled neurons in the posteroventral (PVCN) and anteroventral (AVCN) cochlear nuclei of both sides, which contrasts with the paucity of neurons in the dorsal cochlear nucleus (DCN). **(F)** Widespread distribution of the abundant neurons labeled in the ipsilateral inferior colliculus (IC). **(G)** Small multipolar neurons labeled in the contralateral ventral nucleus of the trapezoid body (VNTB). **(H,I)** Neurons labeled in the locus coeruleus (LC) of both sides. **(J)** Neurons labeled in the ipsilateral ventral tectal longitudinal column (TLCv). **(K,L)** Neurons labeled in the subparafascicular nucleus of the thalamus (SPF) of both sides. **(M,N)** Pyramidal neurons labeled in layer 5 of the auditory cortex of both sides. For other abbreviations, see Figure 1. Calibration bars uncorrected for shrinkage. Calibration bar in **(D)** also applies to **(B,C,E)**. Calibration bar in **(I)** also applies to **(H)**. Calibration bar in **(K)** also applies to **(G,J,L)**. Calibration bar in **(N)** also applies to **(M)**.

The injection site of case FG01 was centered in the ventral portion of the caudal half of SPON. At the level of the maximum tracer spread, the injection site encroached upon all nuclei of the SOC, except for the most medial region of the MNTB and the lateral half of the lateral limb of the LSO (Figure 2A). The rostral third of the SOC was not affected by the injection site.

##### 3.1.1.2. Neurons labeled in nuclei known to innervate the SOC

Abundant neurons were retrogradely labeled in all nuclei known to innervate the SOC, including the anteroventral and posteroventral cochlear nuclei of both sides, the contralateral VNTB, the locus coeruleus of both sides, the ipsilateral IC, the ipsilateral ventral tectal longitudinal column, the subparafascicular nucleus of the thalamus of both sides, and the auditory cerebral cortex of both sides (Figures 2B–N). These observations, which confirm previous descriptions of the sources of input to the rat SOC (Faye-Lund, 1986; Thompson and Schofield, 2000; Mulders and Robertson, 2001; Saldaña et al., 2007, 2009; Gómez-Álvarez and Saldaña, 2016), demonstrate the efficacy of our experiments in labeling the neurons that innervate the injection site.

##### 3.1.1.3. Neurons labeled in the nuclei of the lateral lemniscus (NLL)

Abundant neurons were also labeled in the NLL. To estimate the number of labeled neurons, we first measured the maximum diameter of every neuron with sharp cell body contour labeled in each NLL (Table 1). We then used these values to apply Abercrombie’s correction factor to compensate for oversampling due to split neurons that appear in two adjacent sections (see section “2. Materials and methods”). According to our corrected estimates, the NLL contained 8,481 labeled neurons: 7,115 neurons (83.9% of the total number) on the side of the injection site, and 1,366 neurons (16.1 %) on the contralateral side (Table 2). The appearance of these neurons ranged from faintly labeled cells with a granular, punctate reaction product localized to the cell body and, sometimes, the proximal portion of the primary dendrites, to neurons containing a dense, diffuse reaction product that filled the cell body and dendrites.

**FIGURE 3:**
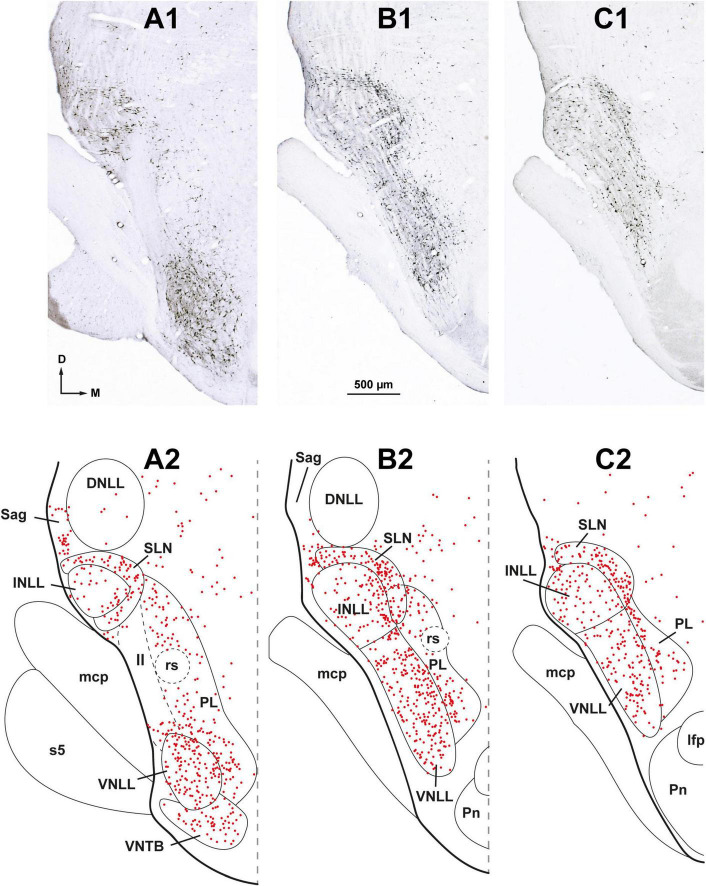
Neurons retrogradely labeled in the nuclei of the lateral lemniscus (NLL) following a large injection of FluoroGold into the ipsilateral superior olivary complex. Case FG01, whose injection site is shown in Figure 2A. **(A1–C1)** Low magnification micrographs of three coronal sections through the NLL. The sections are arranged from caudal to rostral and are regularly spaced by intervals of 320 μm. **(A2–C2)** Plots of the distribution of labeled neurons in the corresponding sections shown above. Each red dot represents one labeled neuron. Abundant labeled neurons are widely distributed throughout the ventral nucleus of the lateral lemniscus (VNLL), the intermediate nucleus of the lateral lemniscus (INLL), the semilunar nucleus (SLN), and the medial paralemniscal region (PL). Notice that the SLN wraps around the INLL. ll, lateral lemniscus; s5, sensory root of the trigeminal nerve. For other abbreviations, see Figure 1. Calibration bar in **(B1)**, uncorrected for shrinkage, applies to all panels.

**FIGURE 4:**
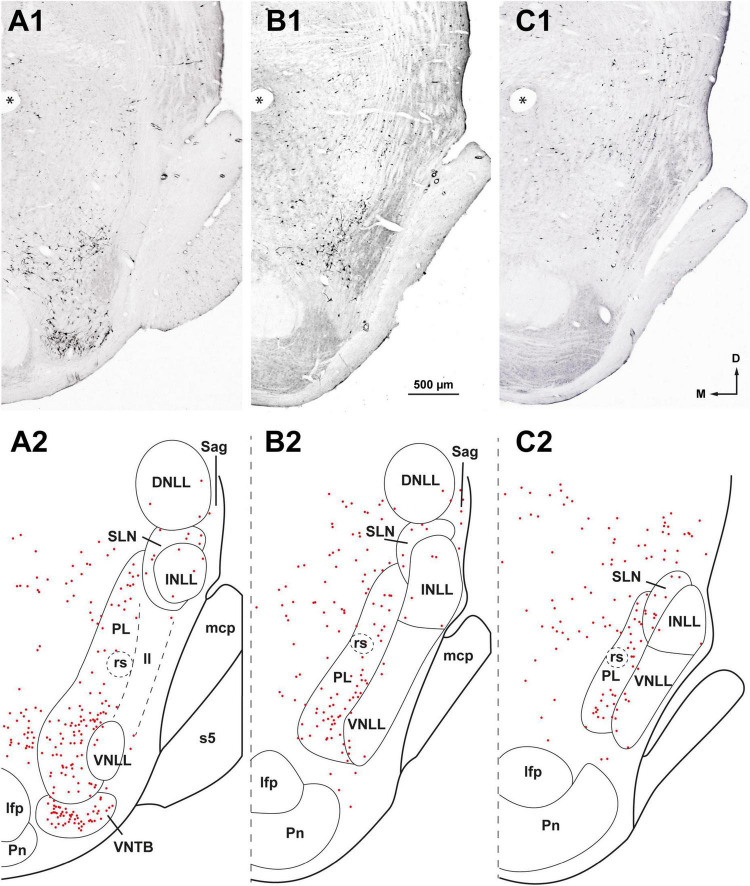
Neurons retrogradely labeled in the nuclei of the lateral lemniscus (NLL) following a large injection of FluoroGold into the contralateral superior olivary complex. Case FG01, whose injection site is shown in Figure 2A. **(A1–C1)** Low magnification micrographs of three coronal sections through the NLL. The sections are ordered from caudal to rostral and are regularly separated by intervals of 320 μm. **(A2–C2)** Plots of the distribution of the labeled neurons in the corresponding sections shown above. Each red dot represents one labeled neuron. Most labeled neurons are found in the medial paralemniscal region (PL); the ventral and the intermediate nuclei of the lateral lemniscus (VNLL, INLL) are almost completely devoid of labeled neurons. The asterisks indicate a fiducial mark used to align the sections dorsoventrally. ll, lateral lemniscus; s5, sensory root of the trigeminal nerve. For other abbreviations, see Figure 1. Calibration bar in **(B1)**, uncorrected for shrinkage, applies to all panels.

Ipsilaterally, most labeled neurons were distributed throughout four of the NLL (Figure 3 and Table 2): the VNLL (2,669 labeled neurons), the INLL (1,255 labeled neurons), the semilunar nucleus (SLN) (1,254 labeled neurons), and the medial paralemniscal region (PL) (1,700 labeled neurons). The nucleus sagulum contained 181 labeled neurons, and they were concentrated in the ventral part of the nucleus. The DNLL contained 55 labeled neurons. Contralaterally, most labeled neurons were found in the PL (928 labeled neurons) and in the SLN (230 labeled neurons) (Figure 4 and Table 2).

In the following paragraphs, we briefly describe the neurons labeled in the NLL. Although the distribution of the labeled neurons differed between the two sides, the neurons labeled contralaterally were similar in size and morphology to those labeled in the corresponding ipsilateral nucleus.

###### 3.1.1.3.1. Neurons labeled in the VNLL

**FIGURE 5:**
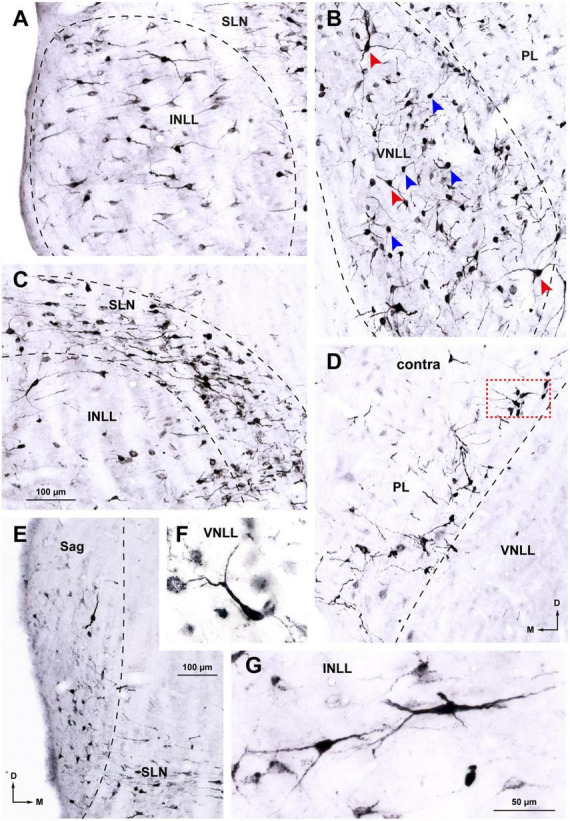
Details of neurons labeled in the ipsilateral **(A–C, E–G)** and contralateral **(D)** nuclei of the lateral lemniscus (NLL) following a large injection of FluoroGold into the superior olivary complex. Case FG01, whose injection site is shown in Figure 2A. Micrographs of coronal sections. **(A)** In the ipsilateral intermediate nucleus of the lateral lemniscus (INLL), labeled neurons are widely distributed. Most of the labeled neurons are multipolar and arranged horizontally. **(B)** In the ventral half of the ipsilateral ventral nucleus of the lateral lemniscus (VNLL), labeled neurons are more densely packed and lack a preferred orientation. Bushy neurons intermingle with multipolar neurons. Blue arrows indicate typical bushy neurons and red arrows indicate typical multipolar neurons. **(C)** In the ipsilateral semilunar nucleus (SLN), most labeled neurons appear flattened and horizontally oriented. Notice that the SLN includes the region dorsal to the INLL, formerly known as the horizontal cell group. The neurons labeled in the SLN show more extensive dendritic filling than those labeled in the INLL. **(D)** In the contralateral medial paralemniscal region (PL), labeled neurons are concentrated near the medial border of the VNLL. Most neurons appear multipolar and lack a preferred dendritic orientation. The boxed area is shown at a higher magnification in Figure 6G. **(E)** In the ipsilateral sagulum (Sag), labeled neurons are predominantly small, and their dendrites show a vertical alignment, in contrast to the horizontal orientation of neurons in the SLN. **(F)** Typical bushy neuron labeled in the ipsilateral VNLL. **(G)** Typical multipolar neurons in the ipsilateral INLL. Calibration bars uncorrected for shrinkage. Calibration bar in **(C)** also applies to **(A,B,D)**. Calibration bar in **(G)** also applies to **(F)**.

In the ipsilateral VNLL, labeled neurons were widely distributed throughout the nucleus without any preferential location (Figures 3, 5B). Most labeled neurons could be classified into two main types: bushy neurons (approximately 60%) and multipolar neurons (approximately 40%).

**FIGURE 6:**
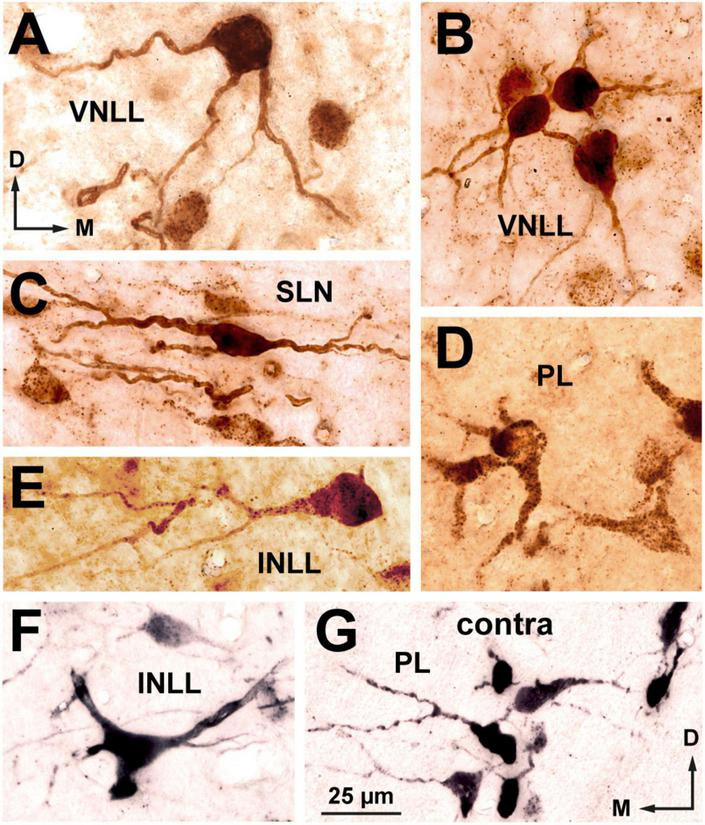
High magnification micrographs of neurons labeled in the ipsilateral **(A–F)** and contralateral **(G)** nuclei of the lateral lemniscus following the injection of FluoroGold into the superior olivary complex. Coronal sections. **(A)** Multipolar neuron of the ventral nucleus of the lateral lemniscus (VNLL). **(B)** Small cluster of bushy neurons from the ventral portion of the VNLL. **(C)** Flat, horizontally oriented neurons from the dorsal part of the semilunar nucleus (SLN). **(D)** Polymorphic neurons from the ventral portion of the ipsilateral medial paralemniscal region (PL). **(E)** A rare bushy-like neuron of the intermediate nucleus of the lateral lemniscus (INLL). **(F)** Multipolar neuron of the INLL. **(G)** Neurons labeled in the contralateral PL. The location of this microscopic field is indicated by the boxed area in Figure 5D. The brown staining in panels **(A–E)** was obtained with a plain peroxidase reaction using diaminobenzidine as the chromogen. In the sections shown in **(F,G)**, the peroxidase reaction was enhanced with nickel salts, hence the black color. The calibration bar in **(G)**, uncorrected for shrinkage, applies to all images.

The bushy neurons of the VNLL resembled morphologically bushy neurons of the ventral cochlear nucleus (Gómez-Nieto and Rubio, 2009; Lauer et al., 2013; Gómez-Álvarez and Saldaña, 2016). They possessed round or oval cell bodies (average maximum diameter = 15.5 μm) and one or, less frequently, two thick, smooth, and sparsely branched primary dendrites that lacked a preferred orientation with respect to the lemniscal fiber bundles (Figures 5F, 6B). Bushy neurons were particularly abundant in the ventral half of the nucleus and became scarcer in progressively more dorsal regions.

Multipolar neurons intermingled with bushy neurons throughout the nucleus (Figure 5B) and were more abundant in the dorsal half of the ipsilateral VNLL. Their cell body appeared triangular or polygonal (average maximum diameter = 19.5 μm) (Figures 5B, 6A). Their relatively thin, tapering dendrites extended over long distances irrespective of the orientation of the lemniscal fibers.

###### 3.1.1.3.2. Neurons labeled in the INLL

Neurons labeled in the ipsilateral INLL were less densely packed than those labeled in the VNLL and showed no preferential distribution (Figures 3, 5A). They were predominantly multipolar (Figures 5A, G, 6F), with an occasional bushy neuron (Figure 6E). Multipolar neurons showed a preferred horizontal orientation of the cell bodies and primary dendrites (Figure 5G), endowing this nucleus with a somewhat stratified appearance (Figure 5A). In coronal sections, the average lateromedial diameter of the cell bodies was 15.9 μm.

###### 3.1.1.3.3. Neurons labeled in the SLN

The SLN surrounds the INLL dorsally, medially, and caudally (Figures 1B, 3). The neurons labeled in the ipsilateral SLN were generally smaller and displayed better dendritic labeling than those in the adjacent INLL (Figure 5C). In the portion of the nucleus that covers the INLL dorsally, separating it from the DNLL, most labeled neurons appeared oriented horizontally, with their dendrites extending medially and laterally (Figures 5C, 6C). The cell bodies were mostly flattened and fusiform, with an average lateromedial diameter of 16 μm and a dorsoventral diameter of 8.2 μm. In the portion of the nucleus covering the medial side of the INLL, the appearance of the labeled neurons was more heterogeneous: although there were a few flat, horizontally oriented neurons, most appeared as small, round cell bodies, 8.2 μm in diameter, with short, truncated dendritic stumps (lower right corner of Figure 5C). This observation suggests that the latter cells are bipolar or multipolar neurons that, due to their rostrocaudally-oriented major axis, had been crosscut. Analysis of variance confirmed that the minimum diameter of the cell body of the horizontal cell group did not differ from the minimum diameter of the neurons labeled medial to the INLL [*F*(1.84) = 2.608, *p* = 0.11], thus suggesting that all SLN neurons belong to the same population.

###### 3.1.1.3.4. Neurons labeled in the PL

On both sides, labeled neurons were widely distributed throughout the PL, with a slight ventral preference (Figures 3, 4). Most labeled neurons were multipolar, but elongated and round cell bodies were occasionally labeled (Figures 5D, 6D, G). Their dendrites showed no preferred orientation. On both sides, the ventral and caudal region of the PL contained well-labeled neurons with a mean cell body diameter of 16.0 μm. In the dorsal half of the PL, the labeled neurons were more scattered, displayed faint labeling and had smaller cell bodies (average maximum diameter = 12.5 μm).

###### 3.1.1.3.5. Neurons labeled in the sagulum

Neurons labeled in the sagulum were concentrated in a territory lateral to the lateral end of the SLN, wedged between the ventrolateral border of the DNLL and the dorsolateral border of the INLL (Figure 5E). Most neurons appeared bipolar or, less frequently, multipolar, with fusiform or triangular cell bodies, with their longest axis oriented vertically, parallel to the brainstem surface. The average maximum diameter of the cell body was 14.7 μm.

###### 3.1.1.3.6. Neurons labeled in the DNLL

The few neurons labeled in the DNLL on both sides displayed the features common to this nucleus: relatively large triangular or multipolar cell bodies (average maximum diameter = 22.5 μm) and predominantly horizontal dendrites.

#### 3.1.2. Cases with smaller injections of FluoroGold into the SOC

**FIGURE 7:**
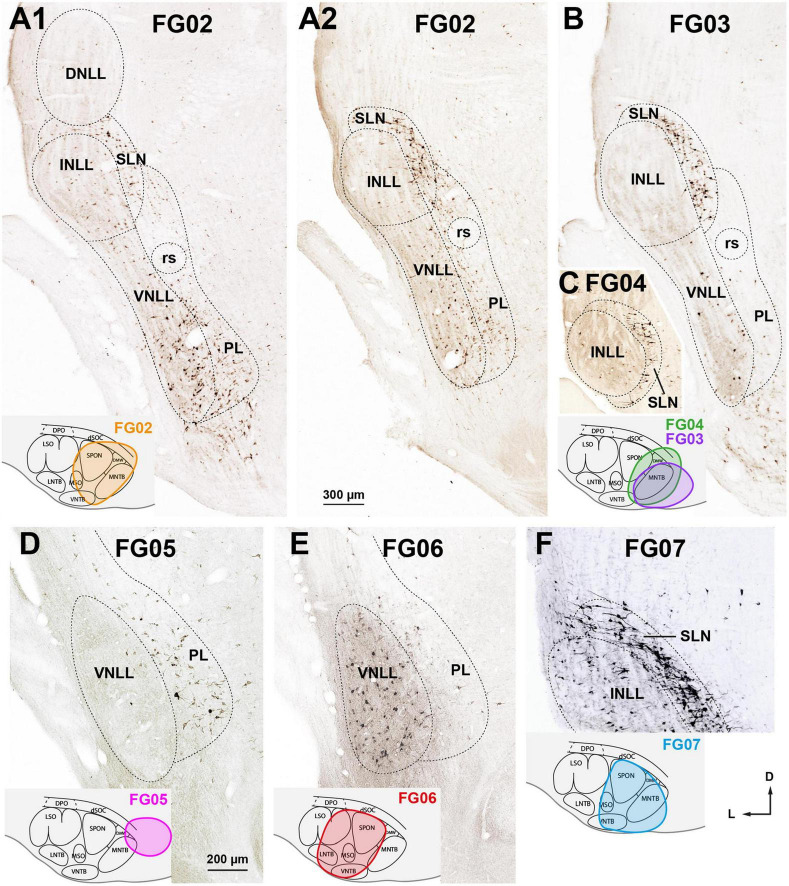
Neurons retrogradely labeled in the nuclei of the lateral lemniscus (NLL) following smaller injections of FluoroGold into the ipsilateral superior olivary complex (SOC). Micrographs of coronal sections. **(A1,A2)** Case FG02. Following a relatively dorsal injection of FluoroGold into the medial half of the SOC (inset), abundant neurons are labeled in the ventral nucleus of the lateral lemniscus (VNLL), the semilunar nucleus (SLN), and the medial paralemniscal region (PL). Section A1 is 160 μm more caudal than section A2. **(B)** Case FG03. Following a very medial injection that mainly affected the medial nucleus of the trapezoid body (MNTB) (inset, purple outline), most labeled neurons were found in the SLN. **(C)** Case FG04. Very caudal section through the intermediate nucleus of the lateral lemniscus (INLL). Notice that the labeled neurons of the SLN surround the INLL. The injection site is shown in the inset, green outline. **(D)** Case FG05. Following a very medial and dorsal injection that affected mostly the dorsal ribbon of the SOC (dSOC) (inset), most labeled neurons were found in the PL, and the VNLL was almost devoid of labeling. **(E)** Case FG06. Following an injection centered in the SOC that spared the dSOC (inset), abundant neurons were labeled in the VNLL, and the PL contained scarce labeled neurons. Notice that the labeling pattern in this case is complementary to that of case FG05, shown in **(D)**. **(F)** Case FG07. Following an injection into the medial half of the SOC that affected mainly the superior paraolivary nucleus (SPON), the ventral nucleus of the trapezoid body (VNTB), and the MNTB (inset), abundant neurons were labeled in the INLL and SLN. For other abbreviations, see Figure 1. Calibration bars uncorrected for shrinkage. Calibration bar in **(A1)** also applies to **(A2,B,C)**, and calibration bar in **(F)** also applies to **(D,F)**.

We analyzed 9 cases whose injection sites of FluoroGold into the SOC were smaller than in case FG01. In these cases, fewer neurons were labeled in the NLL (Table 2), and their distribution depended on the SOC nuclei affected by the injection site. A systematic comparison between cases led to the following conclusion:

1. Following FluoroGold injections into the medial half of the SOC, the distribution and proportions of neurons labeled in all six NLL were similar to those in case FG01 (Table 2). For example, in case FG02, the injection site involved SPON, MNTB, VNTB, MSO, DMW, and dSOC (Figure 7A1 inset) and the labeling pattern (Figures 7A1, A2) was similar to that of case FG01 (Figure 3). Thus, it appears that the medial nuclei of the SOC are innervated by most, if not all, NLL neuron types that send descending projections.

2. Following very medial injections affecting mostly the MNTB (Figure 7B, inset), most labeled neurons were found in the SLN (Figures 7B, C) and to a lesser extent in the VNLL and PL. For instance, in case FG03, whose injection site affected only the MNTB, the SLN contained two thirds of the neurons labeled ipsilaterally (Table 2), and the INLL was almost completely devoid of labeled neurons. Therefore, injecting FluoroGold into the MNTB is an effective procedure to visualize the SLN (Figures 7B, C).
3. Higher proportions of neurons labeled in the PL of both sides were associated with dorsal injection sites involving the dSOC. In case FG05, the injection site affected mostly the dSOC and the dorsomedial tip of the MNTB (Figure 7D, inset), and the PL contained almost 75% of the neurons labeled ipsilaterally (Table 2). The abundance of neurons labeled in the PL contrasted with the paucity of labeling in the VNLL (Figure 7D). This conclusion is strengthened by the comparison between cases FG02 and FG06: although the number of labeled neurons was comparable (Table 2), the proportion of neurons labeled in the PL was much higher in case FG02, whose injection site involved the dSOC (Figure 7A1 inset), than in case FG06, whose injection site, of similar size, spared the dSOC (Figure 7E, inset).
4. Higher proportions of neurons labeled in the INLL were associated with injection sites affecting the VNTB and SPON. Whereas in case FG03, whose injection site spared the VNTB and SPON, the number of neurons labeled in the INLL was negligible, in case FG04, whose injection site encroached upon the VNTB and SPON, the INLL contained 164 labeled neurons (Table 2). Likewise, the percentage of neurons labeled in the INLL was lower in case FG02 than in cases FG06 and FG07, whose injection sites involved more of the VNTB (Table 2 and Figures 7A1, E, F).

### 3.2. Cases with injection of BDA into individual SOC nuclei

**FIGURE 8:**
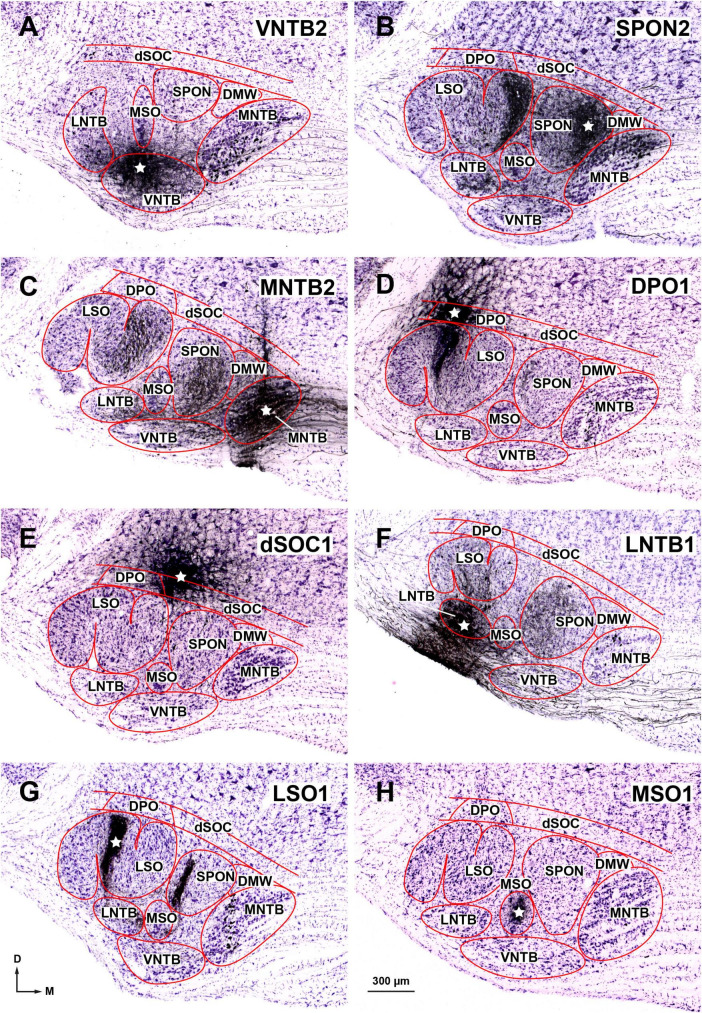
Injection sites of BDA into individual nuclei of the superior olivary complex (SOC). Micrographs of cresyl violet-counterstained, coronal sections through the center of the injection site in: **(A)** The rostral portion of the ventral nucleus of the trapezoid body (VNTB). **(B)** The medial half of the superior paraolivary nucleus (SPON). The dense plexus of labeled fibers in the medial limb of the lateral superior olive (LSO) is formed by collaterals of the axons of MNTB neurons that innervate the injection site (see also Saldaña et al., 2009, their Figures 1A, B; Viñuela et al., 2011, their Figure 1A). **(C)** The caudal portion of the medial nucleus of the trapezoid body (MNTB). **(D)** The dorsal periolivary nucleus (DPO). **(E)** The lateral region of the dorsal ribbon of the SOC (dSOC). **(F)** The caudal region of the lateral nucleus of the trapezoid body (LNTB). **(G)** The central limb of the LSO. The dense vertical plexus of labeled fibers in the SPON is formed by collaterals of the axons of MNTB neurons that innervate the injection site (see also Warr et al., 1997, their Figure 3A). **(H)** The caudal tail of the medial superior olive (MSO). The white stars indicate the center of the injection sites. These and other injection sites are depicted schematically in Figure 11. Other abbreviations: DMW, dorsomedial wedge of the SOC. Calibration bar in **(A)**, uncorrected for shrinkage, applies to all micrographs.

To identify the specific nuclei of the SOC innervated by each NLL, we analyzed qualitatively and quantitatively 47 selected cases with small injections of the bidirectional tracer BDA. In most cases, the injection site was confined to a single SOC nucleus; in the remaining cases, the injection site affected mostly one nucleus and encroached minimally upon neighboring nuclei (Figure 8). Altogether, the BDA experiments explored the sources of projections from the NLL to all regions of the SOC. For each SOC nucleus, we analyzed cases with injections located at different rostrocaudal levels, and the results within each experimental group were similar regardless of the rostrocaudal position of the injection site. In each case, we counted the neurons labeled in the NLL of both sides and analyzed their distribution and morphology.

**FIGURE 9:**
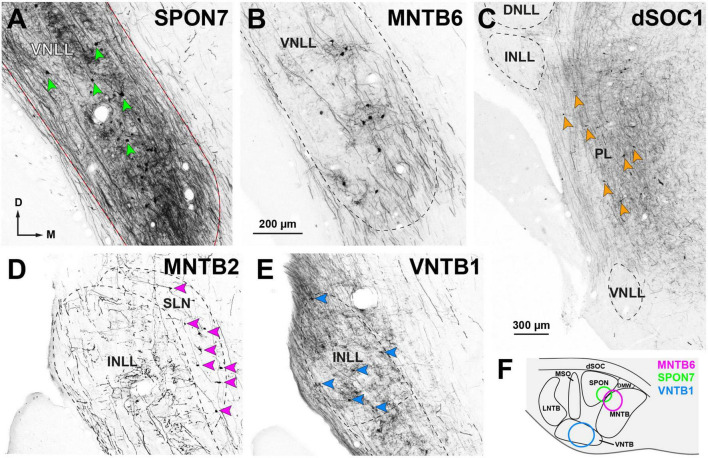
Neurons retrogradely labeled in the nuclei of the lateral lemniscus (NLL) following the injection of BDA into individual nuclei of the ipsilateral superior olivary complex (SOC). Micrographs of coronal sections. **(A)** Case SPON7. Injection into the superior paraolivary nucleus (SPON) resulted in abundant neurons labeled in the ventral nucleus of the lateral lemniscus (VNLL), embedded in a dense plexus of labeled axons. In this and other panels, red arrowheads point to some of the labeled neurons. **(B)** Case MNTB6. The injection into the medial nucleus of the trapezoid body (MNTB) resulted in fewer labeled neurons and axons within the VNLL. **(C)** Case dSOC1. Neurons labeled in the medial paralemniscal region (PL) after injection into the dorsal ribbon of the SOC (dSOC). **(D)** Case MNTB2. Following injection into the MNTB, numerous neurons were labeled in the semilunar nucleus (SLN). The intermediate nucleus of the lateral lemniscus (INLL) contained labeled axons, but no labeled cell bodies. **(E)** Case VNTB1. Injection into the ventral nucleus of the trapezoid body (VNTB) resulted in numerous labeled INLL neurons. **(F)** Schematic representation of the injection sites of the cases depicted in **(A,B,E)**, located in the rostral half of the SOC. The injection sites of the cases depicted in **(C,D)** affected the caudal half of the SOC and are shown in Figure 11. Calibration bar in **(B)**, uncorrected for shrinkage, applies to all panels.

The results of these BDA experiments differed from those of the FluoroGold cases in two main aspects. First, the BDA injection sites were much smaller than the FluoroGold injection sites, so the number of labeled neurons was considerably lower. Second, whereas FluoroGold-labeled neurons appeared stained against an unlabeled background, BDA-labeled neurons were often embedded in a plexus of labeled axons, which did not hinder the characterization of most labeled neurons (Figure 9). The BDA experiments confirmed the results on the morphology and distribution of the NLL neurons labeled with FluoroGold.

A detailed description of the connections of each SOC nucleus is clearly beyond the scope of this account. Therefore, only the neurons labeled retrogradely in the NLL are described below. Representative examples of the anterograde and retrograde labeling obtained in many of the cases included in this study are shown in previous publications (Gómez-Nieto et al., 2008; Saldaña et al., 2009; Viñuela et al., 2011; Gómez-Álvarez and Saldaña, 2016).

**FIGURE 10:**
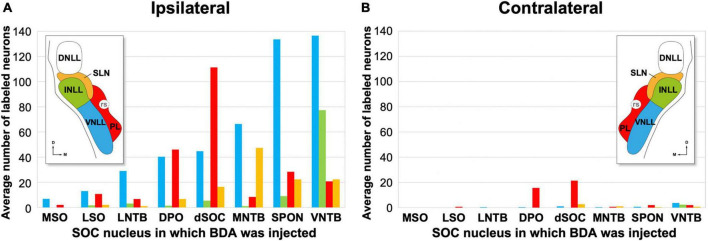
Average number of retrogradely labeled neurons in each nucleus of the lateral lemniscus (NLL) following the injection of BDA into individual nuclei of the superior olivary complex (SOC). For each experimental group, the numbers represent the average values of the three cases with the most labeled neurons; these cases are indicated with asterisks in Table 3. In the histograms, the color of each bar identifies the NLL in which the neurons were labeled, according to the inset. **(A,B)** Illustrate the numbers of neurons labeled ipsilaterally and contralaterally, respectively. For other abbreviations, see Figure 1.

Table 3 shows the number of neurons labeled in each NLL of both sides in our 47 BDA cases, grouped by the SOC nucleus in which the tracer was injected. Figure 10 illustrates the average number of neurons labeled in each NLL for each experimental group, calculated using the three cases of each group with the most labeled neurons. A comparison among cases using the Kruskal–Wallis statistical test revealed that the proportion of neurons labeled in each NLL changed significantly as a function of the SOC nucleus into which BDA was injected (*p* < 0.05). This result demonstrates that each SOC nucleus receives a unique combination of lemniscal inputs.

**TABLE 3:** Number of neurons labeled in each one of the NLL following injections of BDA into individual SOC nuclei.

	Ipsilateral							Contralateral						
Injection site and case number	VNLL	INLL	PL	SLN	Sag	DNLL	Total ipsi	VNLL	INLL	PL	SLN	Sag	DNLL	Total contra
VNTB1*	122	55	10	13	0	1	201	3	1	0	0	0	1	5
VNTB2	104	17	15	16	1	0	153	0	0	0	0	0	0	0
VNTB3	78	31	7	16	0	0	132	0	0	0	0	0	0	0
VNTB4*	178	116	41	40	0	6	381	8	6	6	1	0	2	23
VNTB5*	110	61	11	14	0	1	197	0	0	0	1	0	0	1
VNTB6	87	45	10	13	0	0	155	0	0	0	0	0	1	1
SPON1*	59	4	32	23	0	0	118	0	0	4	0	0	0	4
SPON2*	86	8	25	28	0	1	148	1	0	0	0	0	2	3
SPON3	27	6	12	9	0	0	54	0	0	0	1	0	1	2
SPON4	48	2	12	13	0	0	75	0	0	2	0	0	0	2
SPON5	81	6	11	10	0	0	108	0	0	0	0	0	0	0
SPON6	49	0	2	9	0	0	60	0	0	0	0	0	0	0
SPON7*	256	15	28	16	0	1	316	1	0	2	1	0	3	7
MNTB1*	60	1	5	39	0	0	105	0	0	1	1	0	0	2
MNTB2*	17	0	12	79	0	0	108	0	0	0	0	0	0	0
MNTB3	1	0	2	12	0	0	15	0	0	0	0	0	0	0
MNTB4	61	1	4	7	0	0	73	0	0	0	0	0	0	0
MNTB5	34	6	4	21	0	0	65	0	0	1	0	0	0	1
MNTB6*	122	2	8	24	1	0	157	1	0	1	2	0	0	4
DPO1*	63	1	53	1	0	0	118	0	0	13	0	0	0	13
DPO2	35	2	8	1	0	0	46	0	0	0	0	0	0	0
DPO3*	29	2	35	6	0	0	72	1	0	8	0	0	0	9
DPO4	22	0	44	1	0	0	67	0	0	0	0	0	0	0
DPO5*	29	1	50	13	0	0	93	0	0	26	0	0	0	26
DPO6	63	2	12	1	0	0	78	0	0	1	0	0	0	1
DPO7	38	4	17	5	0	0	64	3	0	1	0	0	1	5
dSOC1*	24	3	127	7	0	0	161	0	0	17	1	0	0	18
dSOC2*	62	8	117	24	0	0	211	0	0	40	5	0	0	45
dSOC3*	48	5	90	18	0	0	161	3	0	7	2	0	0	12
LNTB1*	26	4	8	1	0	0	39	0	0	0	0	0	0	0
LNTB2	3	1	2	2	0	0	8	1	0	0	0	0	0	1
LNTB3*	32	2	5	0	0	0	39	1	0	0	0	0	0	1
LNTB4	10	0	3	0	0	0	13	0	0	0	0	0	0	0
LNTB5	25	0	2	3	0	0	30	0	0	0	0	0	0	0
LNTB6*	29	3	7	2	0	0	41	0	0	0	0	0	1	1
LNTB7	13	0	5	0	0	0	18	0	0	0	1	0	0	1
LSO1	0	0	1	0	0	0	1	0	0	0	0	0	0	0
LSO2	5	1	5	1	0	0	12	2	0	0	0	0	0	2
LSO3	3	0	1	0	0	0	4	0	0	0	0	0	0	0
LSO4*	6	1	7	1	0	0	15	0	0	0	0	0	0	0
LSO5*	12	1	6	0	0	0	19	0	0	0	0	0	0	0
LSO6*	22	3	21	5	0	0	51	0	0	2	0	0	0	2
MSO/LSO1	3	0	6	0	0	0	9	0	0	0	0	0	0	0
MSO1*	1	0	0	0	0	0	1	0	0	0	0	0	0	0
MSO2*	18	0	4	0	0	0	22	0	0	0	0	0	0	0
MSO3*	0	0	1	0	0	0	1	0	0	0	0	0	0	0
DMW1	57	4	15	10	0	0	86	0	0	0	0	0	0	0

*Asterisks indicate the three cases of each experimental group with the most labeled neurons. These three cases were used to calculate the average numbers of labeled neurons depicted in Figure 10.

#### 3.2.1. Cases with injection of BDA into the VNTB

**FIGURE 11:**
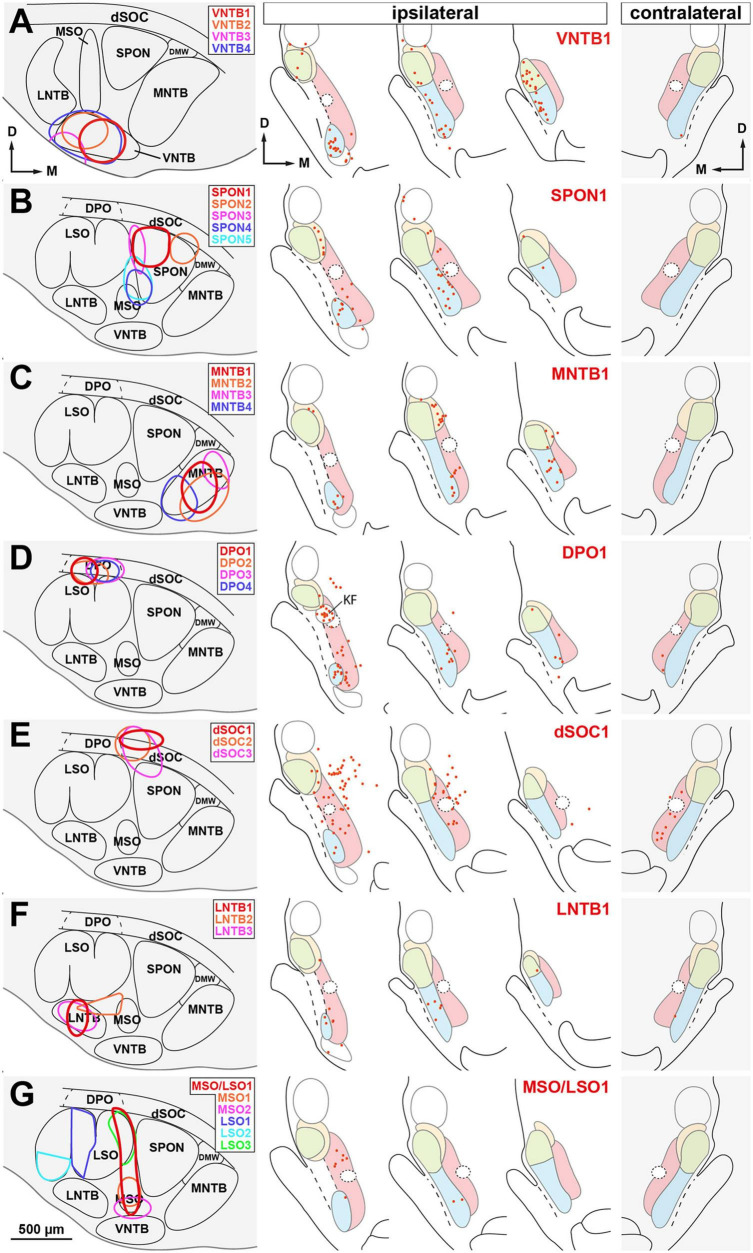
Distribution of the neurons labeled retrogradely in the nuclei of the lateral lemniscus (NLL) following the injection of BDA into individual nuclei of the superior olivary complex (SOC). Cases are segregated in rows according to the SOC nucleus that received the injection site: **(A)** ventral nucleus of the trapezoid body (VNTB); **(B)** superior paraolivary nucleus (SPON); **(C)** medial nucleus of the trapezoid body (MNTB); **(D)** dorsal periolivary nucleus (DPO); **(E)** dorsal ribbon of the SOC (dSOC); **(F)** lateral nucleus of the trapezoid body (LNTB); **(G)** medial and/or lateral superior olive (MSO and/or LSO). In each row, the left panel illustrates schematically the location of several injection sites, identified by colors. The three middle panels of each row show the distribution of the neurons labeled in the ipsilateral NLL in the case whose injection site is depicted in red. The panel on the right shows the distribution of the neurons labeled contralaterally in the same case. Each red dot represents one labeled neuron. Other abbreviations: DMW, dorsomedial wedge of the SOC; KF, Kölliker-Fuse nucleus. Calibration bar in **(G)**, uncorrected for shrinkage, applies to all schemes of the SOC.

These results are based on six representative cases with injections of BDA centered in the VNTB. In most cases, the injection targeted the enlarged, rostrolateral region of the nucleus (Figure 8A). Figure 11A illustrates schematically the location of four injection sites and the distribution of the neurons labeled in the NLL in case VNTB1.

On average, injecting BDA into VNTB resulted in more labeled neurons in the ipsilateral NLL than injecting it into any other SOC nucleus (Table 3 and Figure 10). The contralateral NLL were almost completely devoid of labeled neurons. More than half of the labeled neurons were found in the ipsilateral VNLL. In addition, the INLL contained ten times more labeled neurons in the VNTB cases than in the other cases. These results suggest that the VNTB is the main target of the descending projections from the VNLL and INLL.

#### 3.2.2. Cases with injection of BDA into the SPON

We analyzed seven representative cases with single injections restricted to the SPON. Figures 8B, 11B illustrate the location of five injection sites (see also Saldaña et al., 2009; Viñuela et al., 2011). In the SPON cases, most labeled neurons were found in the ipsilateral VNLL (Figures 10, 11 and Table 3), indicating that the VNLL is the primary source of lemniscal projections to the SPON.

#### 3.2.3. Cases with injection of BDA into the MNTB

We analyzed six representative cases with single injections largely confined to the MNTB. Figures 8C, 11C illustrate the location of the injection sites. On average, the MNTB cases contained many more labeled neurons in the SLN than the other cases (Figures 10, 11 and Table 3), suggesting that the MNTB is the main target of the SLN. Moreover, the MNTB is innervated also by the ipsilateral VNLL.

#### 3.2.4. Cases with injection of BDA into the DPO

These observations are based on seven cases whose injection sites were centered in the DPO, sparing or affecting minimally the underlying LSO. Figures 8D, 11D illustrate the location of four injection sites. On average, the DPO cases contained fewer labeled NLL neurons than the previous cases, and most of them were found in the ipsilateral VNLL and PL (Figures 10, 11 and Table 3).

In the DPO cases, numerous neurons were found in the Kölliker-Fuse nucleus, located medial and caudal to the dorsal third of the VNLL (Figure 11D). The most likely explanation for this result is the involvement within the injection site of the adrenergic group A5, which is located dorsal and lateral to the DPO and receives projections from the Kölliker-Fuse nucleus (Byrum and Guyenet, 1987; Geerling et al., 2017).

#### 3.2.5. Cases with injection of BDA into the dSOC

We analyzed 3 cases with an injection site centered in the lateral half of the dSOC. In case dSOC2, the injection site encroached upon the medial edge of DPO, and in case dSOC3, the tracer spread into the dorsolateral corner of the SPON. Figures 8E, 11E illustrate the location of the injection sites and the distribution of the neurons labeled in case dSOC1. The results of the dSOC cases were unique in two respects: First, these cases contained the largest number of neurons labeled contralaterally. And second, the dSOC cases had the largest number of neurons labeled in the PL of both sides, which was at least four times higher than in the other cases (Table 3). Thus, it appears that the dSOC is the main target of the projections from the PL to the SOC.

#### 3.2.6. Cases with injection of BDA into the LNTB

We analyzed seven cases whose injection site affected only or almost exclusively the LNTB. Figures 8F, 11F illustrate the location of three injection sites in the caudal half of LNTB. Compared to the previous cases, the LNTB cases contained many fewer labeled NLL neurons, and they were found mostly in the ipsilateral VNLL (Table 3).

#### 3.2.7. Cases with injection of BDA into the MSO and/or LSO

We analyzed three cases with single injections of BDA restricted to the MSO, and six representative cases with an injection site restricted to the LSO (see also Gómez-Álvarez and Saldaña, 2016). Our study also included a case whose injection site affected both the MSO and the medial limb of the LSO (case MSO/LSO1). Figures 8G, 11G illustrate the location of six injection sites. Even though numerous neurons were labeled in the nuclei known to innervate the MSO and LSO (including the ipsilateral and contralateral ventral cochlear nucleus, the ipsilateral MNTB, and the contralateral VNTB), the NLL of both sides were almost completely devoid of labeled neurons (Table 3). These findings demonstrate that the MSO and the LSO are unlikely to receive projections from the NLL.

#### 3.2.8. Case with injection of BDA into the DMW

Our collection included only one case whose injection site was centered in the DMW, and the tracer spread into the SPON (not illustrated). Because the results of this experiment were similar to those of SPON cases, no clear conclusions can be drawn about the lemniscal inputs to the DMW.

## 4. Discussion

Our results demonstrate that all nuclei of the rat SOC, except the MSO and LSO, are innervated by abundant NLL neurons. Therefore, the NLL should be regarded as relevant components of the descending auditory system. The principal target of the projections from the VNLL is the VNTB, followed by the SPON, and the MNTB. The INLL appears to innervate selectively the VNTB. The SLN, revealed in this study, innervates preferentially the MNTB. The PL innervates the dSOC of both sides and, to a lesser extent, the DPO. These and other findings are discussed below.

### 4.1. Methodological considerations

The conclusions of our study rest on two methodological pillars: First, we used two reliable neuroanatomical tracers that confirmed and complemented each other’s results. And second, we analyzed in detail a high number of experimental cases. While the large injections of FluoroGold into the SOC revealed the extent to which NLL neurons contribute to descending projections, the focal injections of BDA were instrumental in pinpointing the specific nuclei of the SOC innervated by each NLL.

#### 4.1.1. Control experiments

Because FluoroGold and BDA can be taken up by fibers that cross the injection site without innervating it, we cannot rule out the possibility that some of the neurons revealed in our experiments were the consequence of artifactual tracer uptake by fibers of passage. To evaluate this possibility, we analyzed ten additional archival cases with single injections of FluoroGold or BDA into regions surrounding the SOC. In two cases, BDA was injected caudal to the SOC into the motor nucleus of the facial nerve. In two additional BDA cases, the injection site was centered lateral to the SOC into the principal sensory nucleus of the trigeminal nerve. In two FluoroGold cases and one BDA case, the injection site was located dorsal to the SOC into the caudal pontine reticular formation. In two other cases, BDA was injected dorsolateral to the SOC into the adrenergic group A5. Finally, in one case, BDA was injected dorsomedial to the SOC into the ventral pontine reticular formation. In all these cases, abundant neurons were labeled in the nuclei known to innervate the injection site. In the cochlear nuclei, several cochlear root neurons were labeled in the cases with injections in the motor nucleus of the facial nerve (López et al., 1999). Otherwise, the cochlear nuclei were almost completely devoid of labeled neurons in all cases, demonstrating that the tracers did not spread into the SOC. The NLL of both sides were also devoid of labeled neurons with one notable exception: when the tracers were injected into the reticular formation dorsal to the SOC, there were occasional neurons labeled in the ipsilateral and, to a lesser extent, the contralateral PL. These control experiments support the conclusion that our results were not due to tracer-uptake by fibers that crossed the SOC on their way to more caudal structures, nor to tracer leakage along the pipette tract in territories dorsal to the SOC.

#### 4.1.2. Estimates of the number of labeled neurons

We counted the neurons labeled in the NLL of all cases analyzed. To correct for neurons that were counted twice because they appeared in two adjacent sections, we applied Abercrombie’s correction formula. We chose not to use unbiased stereological methods because in most of our cases, particularly the BDA cases, the number of labeled NLL neurons was too low for adequate stereological sampling.

For several reasons, we presume that our cell counts almost certainly underestimate the number of NLL neurons that innervate the SOC. First, none of our tracer injections affected the entire SOC, not even an entire single SOC nucleus. Most nuclei of the SOC are very elongated in the rostrocaudal direction (Kulesza et al., 2002; Gómez-Álvarez and Saldaña, 2016), and none of our injection sites encompassed the entire longitudinal axis. Second, no matter how efficient, retrograde neuroanatomical tracers never succeed in labeling the entire population of neurons under study (e.g., Lanciego and Wouterlood, 2011). Finally, Abercrombie’s correction leads to an underestimate of the number of cells, because tangentially cut cell caps tend to be overlooked, and Abercrombie’s formula assumes that all neurons have spherical cell bodies, which is not the case (Hedreen, 1998). Because we introduced into the formula the average maximum diameter of the cell body of the neurons labeled in each NLL as the diameter of the particles to be counted, the number of split neurons calculated by the formula was considerably higher than the actual number, thus downsizing the number of labeled neurons.

Despite the limitations of our quantitative analyses, the number of neurons labeled in the NLL was impressive. In our case with the most labeled neurons (case FG01), the sum of neurons labeled in the VNLL and INLL ipsilateral to the injection reached nearly 4,000 neurons. Considering that the rat VNLL and INLL together contain between 12,800 and 13,800 neurons (Kulesza et al., 2002; Ouda and Syka, 2012), our data indicate that at least 30% of the neurons in these two nuclei send their axon to the SOC.

### 4.2. The NLL as players of the descending auditory pathway

The fact that so many NLL neurons are a source of descending projections was an unexpected finding. It may seem surprising that such a prominent group of projections has remained unnoticed until now. Although the reasons for this neglect are unclear, they may be related to the general assumption that most, if not all, neurons in the three classical NLL innervate the IC. This belief is rooted in the impressive labeling of most DNLL, INLL and VNLL neurons following retrograde tracer injections into the IC (e.g., Saint Marie and Baker, 1990; Merchán et al., 1994; Merchán and Berbel, 1996; Kelly et al., 2009).

Previous publications noticed the labeling of NLL neurons following the injection of retrograde tracers into the SOC, which strengthens our current description. For example, the injection of free horseradish peroxidase or peroxidase-conjugated wheat germ agglutinin resulted in “occasional,” “sparse” or “scattered” labeled neurons in the ipsilateral VNLL and INLL (Whitley and Henkel, 1984 [cat]; Glendenning et al., 1985 [cat]; Faye-Lund, 1986 [rat]). Likewise, after the injection of FluoroGold into the SOC, the ipsilateral VNLL and INLL contained “moderate” numbers of labeled neurons (Mulders and Robertson, 2001 [rat]). Interestingly, the mouse VNLL has been reported to contain a few dozen neurons that innervate the cochlea (Suthakar and Ryugo, 2021), thus reinforcing the role of the VNLL in the descending auditory system.

Projections from the NLL to the SOC were also suggested by the results of anterograde tracer experiments. Following injections of [^3^H]leucine into the VNLL and INLL of the cat, anterograde terminal labeling was observed in the ipsilateral SOC, and it was concentrated in dorsal periolivary regions (Kudo, 1981; Whitley and Henkel, 1984).

Our observation of descending projections from the NLL is also supported by a detailed study with intracellular recording and labeling of the physiology and morphology of rat VNLL neurons (Zhao and Wu, 2001). In this study, the axon of two out of nine stellate I neurons and three out of nine stellate III neurons gave rise to descending axonal branches. Although the destination of these axons could not be ascertained due to incomplete filling, the proportion of neurons with ventrally directed axonal collaterals is consistent with our findings.

According to our data, the DNLL does not contribute significant projections to the SOC. Our results differ from those of a previous study, in which the injection of Phaseolus vulgaris-leucoagglutinin (PHA-L) or biocytin into the rat DNLL revealed diffuse projections to all nuclei of the SOC except MSO and LSO (Bajo et al., 1993). Although the reason for this discrepancy remains unknown, the results of Bajo et al. (1993) could be explained if their injection sites affected the DNLL-neighboring SLN and INLL (see their Figure 8C).

Our experiments revealed that numerous PL neurons innervate the dSOC on both sides. Descending projections from paralemniscal regions to dorsal periolivary regions have been previously described using anterograde tracers (Herbert et al., 1997 [rat]; Hannig and Jürgens, 2006 [squirrel monkey]). On the other hand, paralemniscal neurons have been reported to innervate the spinal cord (Liang et al., 2013). Whether the same neurons innervate the dSOC and the spinal cord remains unknown. Although it is possible that our experiments labeled neurons whose axons travel caudally through the dSOC without innervating it, the bilateral labeling of PL neurons in our experiments indicates that the dSOC is indeed a target of the PL.

Although our results have been obtained with rats, the literature cited in the preceding paragraphs suggests that the projections from the NLL to the SOC are shared by a wide variety of mammals. Our own injections of BDA into the SPON of the mouse (Felix et al., 2017) confirm that this nucleus is innervated by numerous VNLL neurons. Similarly, our preliminary injections of retrograde tracers into the SOC of the gerbil and hamster resulted in labeled NLL neurons whenever the injection site involved nuclei other than the LSO and MSO.

### 4.3. The semilunar nucleus (SLN)

Our hodological experiments have revealed a previously unrecognized brain nucleus adjacent to the INLL and DNLL. Because of its appearance in coronal histological sections, we refer to this group of cells as the semilunar nucleus (SLN) of the lateral lemniscus. For a brain territory to be considered a distinct nucleus or subdivision, it must differ from its surrounding structures by at least three main criteria: cytoarchitecture, neural connections, and function (e.g., Kaas, 1982; Saldaña et al., 2007; Aparicio and Saldaña, 2014). The SLN is cytoarchitecturally distinct. It is composed of neurons whose cell bodies are considerably smaller and more tightly packed than those of the adjacent INLL and DNLL. SLN neurons tend to be flat and arranged horizontally. This feature had been noted in previous investigations, which referred to the dorsal portion of the SLN, sandwiched between the INLL and DNLL, as the “horizontal cell group” (Caicedo and Herbert, 1993). Our results demonstrate that the flat neurons of the SLN cover the INLL not only dorsally, but also medially and caudally.

The difference between the SLN and the INLL and DNLL is readily apparent in sections immunostained for parvalbumin, in which the immunonegative SLN contrasts with the strongly stained INLL and DNLL (Seto-Ohshima et al., 1990 [gerbil]; Vater and Braun, 1994 [horseshoe bat]; Caicedo et al., 1996 [guinea pig]; Lohmann and Friauf, 1996 [rat]; Paxinos et al., 1999 [rat]; Budinger et al., 2000 [gerbil]; Spencer et al., 2002 [rat]). Likewise, the INLL and DNLL, but not the SLN, are stained with histochemical methods for cytochrome oxidase or for Wisteria floribunda agglutinin (Benson and Cant, 2008 [gerbil]). A complementary image is obtained in sections immunostained for calbindin, in which the positive cell bodies of the SLN delineate the dorsal and medial borders of the faintly stained INLL (Caicedo et al., 1996 [guinea pig], compare their Figure 10C with our Figure 7C).

The identity of the SLN is further strengthened by the expression of several molecular markers. Among the genes that are strongly expressed in the SLN, but not in the neighboring nuclei, are those encoding the theta subunit of the GABA_A_ receptor (Babrq), the CAM kinase-like vesicle-associated protein (Camkv), the neuronal guanine nucleotide exchange factor (Ngef), or the glutathione peroxidase 3 (Gpx3) (Allen Brain Atlas).^1^

The SLN is also hodologically distinct. Although nothing is known about the sources of ascending inputs to the SLN, this nucleus receives direct projections from the primary auditory cortex, which does not innervate either the DNLL or the INLL [reviewed by Saldaña (2015)]. Furthermore, our results demonstrate that SLN neurons are labeled selectively following injections of retrograde tracers into the ipsilateral MNTB, thus revealing a previously unknown SLN-to-MNTB projection. In mice, gerbils, and bats, axons of unknown origin descend in the lateral lemniscus to target MNTB (Kuwabara et al., 1991), an observation coherent with our results.

The function of the SLN is unknown. However, several lines of research suggest that SLN neurons are glutamatergic and, therefore, excitatory. First, the SLN lacks neurons immunoreactive for GABA or glycine (Riquelme et al., 2001). Second, SLN neurons do not express the mRNA for glutamic acid decarboxylase (GAD), the enzyme that synthetizes GABA (Riquelme et al., 2001; Tanaka and Ezure, 2004). And three, SLN neurons do not express the mRNA for the vesicular inhibitory amino acid transporter (VIAAT), which is used by GABAergic and glycinergic neurons, nor the mRNA for the glycine transporter 2 (GLYT2), which is characteristic of glycinergic neurons; instead, most SLN neurons express the mRNA for the type 2 vesicular glutamate transporter (VGLUT2), which is used by a subset of glutamatergic neurons (Tanaka and Ezure, 2004; Ito et al., 2011).

Taken together, the neurochemical data and our tract-tracing results suggest that the SLN may constitute a novel source of excitation for MNTB neurons, and presumably the first identified descending input to this nucleus. Indeed, our preliminary experiments with injection of an anterograde tracer into the SLN confirm the novel projection and show that the axons that innervate MNTB are thin and bear small terminal and en passant boutons that end in close apposition to the cell body of principal MNTB neurons or in the neuropil. This pattern of innervation is consistent with the non-calyceal inputs that innervate the MNTB, which account for 5% of the synaptic coverage of the cell body (Smith et al., 1991). These non-calyceal inputs can trigger postsynaptic action potentials with a longer latency and lower reliability than the larger calyceal input from globular bushy cells (Hamann et al., 2003). Although the source of these non-calyceal inputs has remained elusive (Smith et al., 1991; Hamann et al., 2003; Milinkeviciute et al., 2019), the SLN presents itself as a strong candidate.

One may wonder about the relationship between the SLN and the so-called medial paralemniscal nucleus, located dorsal to the rubrospinal tract and medial to the INLL (Varga et al., 2008). While the location of the medial paralemniscal nucleus seems partly coincident with that of our SLN, the anatomical and hodological features of these nuclei suggest that they are two separate structures. The main distinguishing feature of the medial paralemniscal nucleus is that many of its neurons express the tuberoinfundibular peptide of 39 residues (TIP39) (Dobolyi et al., 2002, 2003a,b; Faber et al., 2007; Varga et al., 2008). The territory occupied by the TIP39-immunopositive neurons is wider lateromedially, and shorter dorsoventrally, than the SLN. The TIP39-immunopositive neurons do not reach as far dorsally as the dorsal border of the INLL, which is covered by the horizontally oriented neurons of the SLN. In addition, the lateral border of the medial paralemniscal nucleus seems to stay at a distance from the medial border of the INLL (see Dobolyi et al., 2003a, their Figure 8; Varga et al., 2008, their Figure 2A2), whereas the SLN wraps tightly around it. Furthermore, the medial paralemniscal nucleus consists of a mixture of small and large neurons, whereas the SLN is composed of a homogeneous population of small, flattened neurons. Finally, the MNTB lacks TIP39-immunoreactive fibers (Dobolyi et al., 2003b, their Figure 10F), but it is innervated by the SLN. The identity of the medial paralemniscal nucleus and the SLN could be resolved with experiments that combine immunostaining of the medial paralemniscal nucleus for TIP39 with labeling of the SLN with a retrograde tracer injected into the MNTB.

### 4.4. Functional implications

Our results have unraveled a number of pathways that arise from multiple NLL and target multiple nuclei of the SOC. Therefore, it would be unrealistic to ascribe a single or common function to all of these projections. Because the lemniscal projections spare the LSO and MSO, they are unlikely to be involved in sound localization.

The projections described herein are unique in at least two aspects. On the one hand, the projections of many VNLL and INLL neurons exhibit a degree of divergence not seen at other levels of the auditory pathway, because, in addition to innervating the SOC, they also innervate the IC. On the other hand, the descending NLL projections are neurochemically heterogeneous. Whereas the projections of the INLL and SLN are presumably excitatory, most VNLL neurons are inhibitory. Thus, the descending projections of the VNLL differ from the other descending afferents to the SOC, which originate from the IC and auditory cortex and are excitatory (Schofield and Beebe, 2018).

The VNTB is innervated by more NLL neurons than any other SOC nucleus. This finding reinforces the role of the VNTB as a major hub in the descending auditory pathway, as this nucleus is also the main SOC target of projections from the IC (Caicedo and Herbert, 1993; Thompson and Thompson, 1993; Vetter et al., 1993; Malmierca et al., 1996) and the auditory cortex (Feliciano et al., 1995; Doucet et al., 2002; Coomes and Schofield, 2004). Six VNTB neuron types have been identified based on the primary target of their projections: the cochlea (White and Warr, 1983; Aschoff and Ostwald, 1987; Vetter and Mugnaini, 1992; Mulders and Robertson, 2000), the IC (Faye-Lund, 1986; Aschoff and Ostwald, 1988), the superficial layers of the dorsal cochlear nucleus (DCN) (Warr and Beck, 1996), the cochlear root neurons (Gómez-Nieto et al., 2008, 2014), the ipsilateral MNTB (Albrecht et al., 2014), and the contralateral LSO (Warr and Beck, 1996; Gómez-Álvarez and Saldaña, 2016). The specific neuron types of the VNTB innervated by the NLL are unknown. However, indirect evidence suggests that the targets of the VNLL and INLL include medial olivocochlear neurons. Following the cochlear injection of the Bartha strain of pseudorabies virus (PRV), which causes retrograde transneuronal labeling, both lateral and medial olivocochlear neurons were initially labeled. With longer survival times, labeled cell bodies were found in the NLL (Horváth et al., 2003; Brown et al., 2013). Since the NLL do not innervate the LSO, the most likely explanation for this observation is that the labeled NLL neurons contact medial olivocochlear neurons. Moreover, the fact that more NLL neurons were transsynaptically labeled on the side contralateral to the injection site (Brown et al., 2013) suggests that the projections from the NLL contact preferentially crossed medial olivocochlear neurons.

The SPON is also innervated by a considerable number of NLL neurons. This finding is relevant, because the rat SPON does not receive substantial direct projections from either the IC (Faye-Lund, 1986; Caicedo and Herbert, 1993; Vetter et al., 1993) or the auditory cortex (Feliciano et al., 1995). Thus, the input from the NLL adds to other descending projections to the SPON, which originate in the ventral tectal longitudinal column (Viñuela et al., 2011) and in the subparafascicular nucleus (Yasui et al., 1992). Taken together, these data suggest that the NLL may be the lowest level of the auditory pathway that provides top-down feedback to the SOC.

In summary, our study unravels prominent projections from the NLL to the SOC, which will have to be further characterized neurochemically and with anterograde tracers in future studies. Each SOC nucleus, except LSO and MSO, receives a unique combination of inputs from the NLL. The previously unknown SLN may provide the first identified non-calyceal excitatory input to MNTB neurons. Based on the proportion of NLL neurons involved in all these projections, the NLL should be considered major characters in the descending auditory pathway.

## Data availability statement

The raw data supporting the conclusions of this article will be made available by the authors, without undue reservation.

## Ethics statement

The animal study was reviewed and approved by the Bioethics Committee of the University of Salamanca.

## Author contributions

ES: study concept and design, obtained funding, and study supervision. MG-M, HR, MG-Á, RG-N, and ES: acquisition of data and critical revision of the manuscript for important intellectual content. MG-M, HR, and ES: analysis and interpretation of data and drafting of the manuscript. All authors had full access to all the data in the study and took responsibility for the integrity of the data, the accuracy of the data analysis, and approved the submitted version.

## Footnotes

1
https://mouse.brain-map.org/
